# Prolyl oligopeptidase inhibition reduces alpha‐synuclein aggregation in a cellular model of multiple system atrophy

**DOI:** 10.1111/jcmm.16910

**Published:** 2021-09-05

**Authors:** Hengjing Cui, Tommi Kilpeläinen, Lydia Zouzoula, Samuli Auno, Kalevi Trontti, Sampo Kurvonen, Susanna Norrbacka, Iiris Hovatta, Poul Henning Jensen, Timo T. Myöhänen

**Affiliations:** ^1^ Division of Pharmacology and Pharmacotherapy/Drug Research Program University of Helsinki Helsinki Finland; ^2^ SleepWell Research Program Faculty of Medicine University of Helsinki Helsinki Finland; ^3^ Department of Psychology and Logopedics University of Helsinki Helsinki Finland; ^4^ Neuroscience Center University of Helsinki Helsinki Finland; ^5^ Department of Biomedicine & DANDRITE Aarhus University Aarhus Denmark; ^6^ Integrative Physiology and Pharmacology Unit Institute of Biomedicine University of Turku Turku Finland; ^7^ School of Pharmacy University of Eastern Finland Kuopio Finland

**Keywords:** alpha‐synuclein, KYP‐2047, multiple system atrophy, neurodegeneration, prolyl oligopeptidase, synucleinopathies

## Abstract

Multiple system atrophy (MSA) is a fatal neurodegenerative disease where the histopathological hallmark is glial cytoplasmic inclusions in oligodendrocytes, rich of aggregated alpha‐synuclein (aSyn). Therefore, therapies targeting aSyn aggregation and toxicity have been studied as a possible disease‐modifying therapy for MSA. Our earlier studies show that inhibition of prolyl oligopeptidase (PREP) with KYP‐2047 reduces aSyn aggregates in several models. Here, we tested the effects of KYP‐2047 on a MSA cellular models, using rat OLN‐AS7 and human MO3.13 oligodendrocyte cells. As translocation of p25α to cell cytosol has been identified as an inducer of aSyn aggregation in MSA models, the cells were transiently transfected with p25α. Similar to earlier studies, p25α increased aSyn phosphorylation and aggregation, and caused tubulin retraction and impaired autophagy in OLN‐AS7 cells. In both cellular models, p25α transfection increased significantly aSyn mRNA levels and also increased the levels of inactive protein phosphatase 2A (PP2A). However, aSyn or p25α did not cause any cellular death in MO3.13 cells, questioning their use as a MSA model. Simultaneous administration of 10 µM KYP‐2047 improved cell viability, decreased insoluble phosphorylated aSyn and normalized autophagy in OLN‐AS7 cells but similar impact was not seen in MO3.13 cells.

## 1 INTRODUCTION

Multiple system atrophy (MSA) is a sporadic, adult‐onset and progressive neurodegenerative disease with a prevalence of 0.1‐3/100,000 persons/year that increases with ageing.^1^ Since current therapies are only symptomatic, MSA is a fatal disease with survival duration of 6–9 years after the onset of symptoms. According to the predominant motor disorder, MSA is categorized as MSA‐P or MSA‐C.^1^ Both categories have serious autonomic failure but MSA‐P is described mostly by parkinsonism due to the prevailing striatonigral degeneration and MSA‐C is characterized by cerebellar ataxia due to olivopontocerebellar atrophy.^1^

The main histopathological hallmark of MSA is the prevalence of glial cytoplasmic inclusions (GCIs) in oligodendrocytes.^2^ In 1998, Wakabayashi et al.^3^ detected that α‐synuclein (aSyn) is the main component of GCIs, categorizing MSA to the synucleinopathies with Parkinson's disease (PD) and dementia with Lewy bodies (DLB). Unlike in PD and dementia with Lewy bodies, where aSyn is accumulated mostly in neurons, in MSA aSyn is accumulated in the cytoplasm of oligodendroglial cells.^3^ aSyn in GCIs is mostly present in a fibrillary oligomeric form^4^ and as serine 129 phosphorylated (pS129 aSyn) that is considered to destabilize aSyn and elevate its aggregation.^5^ aSyn oligomerization has been shown to cause loss‐of‐function toxicity, for example in synaptic vesicle regulation, and damage several cellular organelles, including endoplasmic reticulum, mitochondria and protein degradation pathways (for review see^6^). Animal models of MSA have shown that aSyn overexpression in oligodendrocytes causes cellular death, leading to demyelination, neuronal damages and premature death,^7^, ^8^ suggesting the involvement of aSyn in the pathophysiology of MSA.

It has been under debate if aSyn in GCIs is exogenous and transferred from neurons by cell‐to‐cell transfer since only low amounts of aSyn mRNA have been measured in healthy and MSA patient‐derived oligodendrocytes.^9^ However, recent studies suggest that endogenous aSyn in oligodendrocytes is critical for aSyn aggregation in MSA after the exogenous aSyn has triggered the process.^10^ Another important factor contributing to aSyn aggregation in MSA is p25α/TPPP protein (tubulin polymerization‐promoting protein; p25α). p25α is a protein promoting tubulin assembly and maintaining myelin sheath^11^ but in MSA it relocates from myelin sheaths and accumulates to oligodendroglial soma.^12^ This redistribution is associated with demyelination of axons, and subsequently, phosphorylation and aggregation of aSyn, leading to the abovementioned aggregation process.

Compounds having an effect on aSyn aggregation or degradation, such as anle138b^13^ or enhancement of aSyn degradation by autophagy activation,^14^ respectively, have shown beneficial effects on experimental MSA models. Therefore, targeting aSyn could be a potential disease‐modifying therapy for MSA. In our previous studies, we have shown that a serine protease, prolyl oligopeptidase (PREP), increases aSyn oligomerization via direct protein‐protein interaction^15^, ^16^ and negatively regulates autophagy.^17^ Importantly, small‐molecular PREP inhibitors can interfere with this interaction, leading to decreased aSyn aggregation,^15^, ^18^, ^19^ and PREP inhibition or deletion also stimulates autophagy and increases the degradation of aSyn oligomers^16^, ^17^, ^18^ and fibrils.^20^ In aSyn‐based *in vivo* PD model, PREP inhibition has shown disease‐modifying impact by restoring an aSyn virus vector‐induced behavioural deficit.^21^ Therefore, we tested if PREP inhibition has a beneficial effect on aSyn accumulation and toxicity in a cellular model of MSA.

## 2 MATERIALS AND METHODS

### 2.1 Reagents

Reagents were purchased from Sigma‐Aldrich (St Louis, MO) if not otherwise specified. Ethanol was purchased from Altia (Helsinki, Finland). The PREP inhibitor, KYP‐2047 (4‐phenylbutanoyl‐l‐prolyl‐2(S)‐cyanopyrrolidine), was synthesized by us as described earlier.^22^

### 2.2 Cell cultures

#### 2.2.1 OLN‐AS7 cell culture

OLN‐AS7 cells^23^ expressing human aSyn were used in this study. OLN‐AS7 originate from immortalized rat oligodendrocyte OLN‐93 cell culture that are transfected with a pcDNA3.1 zeo(−) aSyn vector (see^23^ for more detailed description). OLN‐AS7 cells were maintained at +37°C under 5% CO_2_ and grown in full Eagle's medium (DMEM; #D6429) with an additional 10% (v/v) foetal bovine serum (FBS; #16000‐044, Thermo Fisher Scientific), 1% (v/v) penicillin‐streptomycin solution (#15140122, Thermo Fisher Scientific) and 100 μg/ml zeocin (R25005, Thermo Fisher Scientific). For experiments, cells were seeded without zeocin 24 h before treatment. Cells were used for experiments in passages 3–15.

#### 2.2.2 MO3.13 cell culture

The human oligodendrocyte cell line, MO3.13 (Cedarlane), was obtained from tebu‐bio (Roskilde, Denmark), and grown in high glucose Dulbecco's modified Eagle's medium (DMEM) (DMEM‐Glutamax; #31966021, Thermo Fisher Scientific) with 10% foetal bovine serum (FBS; #16000‐044, Thermo Fisher Scientific) and 1% (v/v) penicillin‐streptomycin solution (#15140122, Thermo Fisher Scientific) in a humidified incubator with 5% CO_2_ at 37°C. Prior to treatment, cells were seeded in 6‐well plates with 4 × 10^5^ cells/well density and incubated for 24 h. Cells were used for experiments in passages 3–15.

### 2.3 Cell treatments

p25α plasmid transfections in OLN‐AS7 cells were done using Lipofectamine 3000 (L3000015; Thermo Fisher Scientific) according to the manufacturer's instructions 24 h prior to the treatments. Production of pcDNA3.1 zeo(‐) plasmid expressing human p25α is presented in.^24^ For MO3.13 cells, p25α plasmid transfection was performed similar to OLN‐AS7 cells, and for aSyn transfection, AAV1‐EF1a‐V5‐synuclein (Addgene #60057; Dr. Brandon Harvey, Intramural Research Program, National Institute on Drug Abuse, Baltimore, MD). aSyn plasmid construction was described in.^17^ pAAV‐EF1a control vector with 50 bp insert was created as described in.^25^ aSyn plasmid concentration was kept similar when transfected with empty plasmid or in combination with p25α to avoid changes in protein expression, and empty plasmid was used also with p25α transfection. For PREP inhibitor experiments, KYP‐2047 was diluted to cell culture medium from 100 mM stock in 100% DMSO to 1 and 10 µM, and corresponding concentration of DMSO was used as a vehicle control.

### 2.4 PREP enzyme activity assay

For the PREP activity assay, fluorometric assay based on Suc‐Gly‐Pro‐aminomethylcoumarin (AMC) substrate was used as in Myöhänen et al. (2012).^26^ Briefly, cells were plated to 6‐wellplate with density of 400,000 cells/well, transfected with p25α and after 24 h, lysed with a lysis buffer. PREP activity was measured from supernatants, and released AMC was correlated on total protein amounts measured by using bicinchoninic acid method (BCA; Pierce BCA Protein Assay Kit, #23225, Thermo Fisher Scientific). All activity measurements were made in triplicate with 2 biological repeats.

### 2.5 Cell viability assay

OLN‐AS7 and MO3.13 cells were plated with the density of 10,000 cell/well in 96‐well plate and the next day transfected by p25α (OLN‐AS7) or with p25α and aSyn (MO3.13). 24 h after transfection, the cells were incubated for 48 h in the presence of 1 μM or 10 µM KYP‐2047 or DMSO vehicle (0.01% DMSO; 150 μl/well). LDH release assay was performed as previously described.^26^

### 2.6 Immunocytochemistry

Immunocytochemistry (ICC) was used to detect changes in total aSyn, pS129 aSyn and tubulin retraction after p25α transfection and PREP inhibition. Briefly, OLN‐AS7 cells were plated over glass coverslips in a 12‐well plate with a density of 60,000 cells/well and allowed to attach overnight. 24 h after p25α transfection, cells were treated with 10 µM KYP‐2047 for 48 h and finally fixed with 4% paraformaldehyde for staining. 10% normal goat serum (S‐1000, Vector Laboratories) was used for blocking for 30 min and thereafter cells were incubated with primary antibodies against aSyn, pS129 aSyn, α‐tubulin or p25α overnight at +4°C (details in Table 1). After washes, the following secondary antibodies were used to incubate cells 2 h in room temperature: for sheep aSyn, anti‐sheep Alexa Fluor488 (1:400; ab150177, Abcam; RRID: AB_2801320); for mouse pS129 aSyn, anti‐mouse Alexa Fluor488 (1:800; ab150113, Abcam; RRID: AB_2576208); for rabbit p25α, anti‐rabbit Alexa Fluor 568 (1:800; ab175471, Abcam; RRID: AB_2576207). α‐tubulin antibody was conjugated with Alexa Fluor 488, and no secondary antibody incubation was used. Cells were mounted with Vectashield containing DAPI to stain nuclei (H‐1200, Vector Laboratories; RRID: AB_2336790). Imaging was performed using Leica TCS SP5 confocal microscope (Leica Microsystems). In tubulin retraction analysis, images were converted to 8‐bit grayscale format, and the area of tubulin network in p25α transfected cells was analysed by ImageJ (version 1.51; National Institute of Health; RRID:SCR_002285) with a protocol described in.^23^ For publication purposes, the images were converted from grayscale to RGB colour and recoloured with corresponding colours, and minor modifications to brightness and contrast were made. All modifications to brightness and contrast were done similarly to each image, and they were analysed prior to modifications.

**TABLE 1:** Details of antibodies used

Antigen	Species	Manufacturer	Product #/RRID	Dilution
aSyn (ICC and WB)	Sheep	Abcam	ab6162/AB_2192805	1:1000
pS129 aSyn (ICC)	Mouse	Abcam	ab184674/AB_2819037	1:400
pS129 aSyn (WB)	Rabbit	Abcam	ab51253/AB_869973	1:500
p25α (ICC)	Rabbit	Abcam	ab92305/AB_2050408	1:1000
α‐tubulin (ICC)	Mouse	Abcam	ab195887	1:200
PP2AC(α + β); Clone Y119	Rabbit	Abcam	ab32141/RRID:AB_2169649	1:2000
PP2A phospho‐Tyr307	Rabbit	Thermo Fisher	PA5‐36874/RRID:AB_255379	1:500
LC3B (WB)	Rabbit	Sigma	L7543/AB_796155	1:1000
SQSTM_1_/p62 (WB)	Mouse	Abcam	ab56416/AB_945626	1:5000
β‐actin (WB)	Rabbit	Abcam	ab8227/AB_2305186	1:2000
Vinculin (WB)	Rabbit	Abcam	ab129002/AB_11144129	1:10000

Abbreviations: ICC, immunocytochemistry; WB, Western blot.

### 2.7 Western blot

For Western blot (WB) assays, 200,000 cells were seeded in 6‐well plates, transfected with p25α and treated with 10 µM KYP‐2047 for 24 or 48 h. Thereafter, the cells were lysed in ice‐cold RIPA buffer supplemented with Halt Phosphatase (#87786, Thermo Fisher Scientific) and Protease Inhibitor cocktails (#78430, Thermo Fisher Scientific), sonicated with ultrasonicator and then centrifuged at 14,000 *g* for 60 min.^17^, ^26^ Supernatant was collected as the soluble fraction, and the pellet was dissolved in SDS‐containing Laemmli buffer as an SDS‐soluble and insoluble fraction.^17^, ^26^ Protein concentration was measured from soluble fraction by BCA. 30 µg of sample was loaded to 12% stain‐free Mini‐Protean TGX gels (#4568044, Bio‐Rad) for SDS‐PAGE. Gels were transferred by Trans‐Blot Turbo Transfer System (#1704150, Bio‐Rad) onto Trans‐Blot Turbo Midi PVDF (#1704157, Bio‐Rad) membrane. For insoluble fraction, total protein amount was visualized from stain‐free gel by using the ChemiDoc XRS+ (Bio‐Rad). Thereafter, the membranes for aSyn and pS129 aSyn immunoblotting were incubated 30 min in 4% paraformaldehyde for fixing. The membranes were blocked with 5% skim milk and incubated in primary antibodies (Table 1) at +4ºC overnight. The following HRP‐conjugated secondary antibodies were used: goat anti‐rabbit (1:2000; #31463; Thermo Fisher Scientific) for pS129 aSyn, vinculin, β‐actin and LC3B; donkey anti‐sheep HRP (1:2000, ab6900, Abcam; RRID:AB_955452) for total aSyn; goat anti‐mouse HRP (1:5000; #31430; Thermo Fisher Scientific; RRID:AB_228307) for p62. The images were captured using the ChemiDoc XRS+ (Bio‐Rad).

To verify that bands are in the linear range of detection, increasing exposure time and automatic detection of saturated pixels in ImageLab software (version 6.01, Bio‐Rad) was used. For analysis, images were converted to 8‐bit grayscale format, and the optical densities (OD) of the bands were measured by ImageJ (histogram area analysis). The OD obtained from each band was normalized against the corresponding β‐actin (OLN‐AS7 cells) or vinculin band (MO3.13 cells), as in our earlier studies.^16^, ^25^ All immunoblotting analyses were done with 3–4 technical replicates of each treatment per membrane and with 2–3 biological replicates (at least 3 different WB assays). Full blot images are presented in Supplementary files.

### 2.8 Quantitative real‐time PCR

250 ng of DNase I (Thermo Scientific)‐treated total RNA was converted to cDNA with iScript select cDNA synthesis kit (Bio‐Rad Laboratories) and amplified with 250 nM of primers in CFX384 Real‐Time PCR cycler using IQ SYBR Green Supermix (Bio‐Rad Laboratories). Following primers were used for aSyn (*Snca*): forward, AGGACTTTCAAAGGCCAAGG; reverse, TCCTCCAACATTTGTCACTTGC.^26^ Expression levels were normalized to *Actb* (MO3.13 cells: forward GATTCCTATGTGGGCGACGA, reverse ATAGCACAGCCTGGATAGCA, OLN‐AS7 cells: forward, ACCCTAAGGCCAACCGTGAA; reverse, TGCTCGAAGTCTAGGGCAAC). Each reaction was run in triplicate, and relative expression level was calculated using a standard curve (7.15, 10.0, 5.0, 2.0, 1.0, 0.5 and 0.25 ng of cDNA) with CFX Manager (Bio‐Rad Laboratories).

### 2.9 Statistical analyses

All experiments were done at least in triplicate, and samples were not used to re‐analyse the same protein. Data are expressed as mean values ±standard error of the mean (mean ± SEM), and negative control average was set as 100% on each assay to reduce variability between repeats. Error bars in the figures represent SEM if not otherwise stated in the figure legend. Differences between two groups were analysed using two‐tailed unpaired Student's *t‐*test. For more than 2 groups, 1‐way analysis of variance (ANOVA) was followed by Tukey's post hoc comparison if ANOVA assay gave statistical significance (*p *< 0.05). In all cases, *p *< 0.05 were considered to be significant. Statistical analysis was performed using PRISM GraphPad statistical software (version 6.07, GraphPad Software, Inc.).

## 3 RESULTS

### 3.1 PREP inhibition reduces p25α induced cell death and tubulin retraction in OLN‐AS7 cells

**FIGURE 1:**
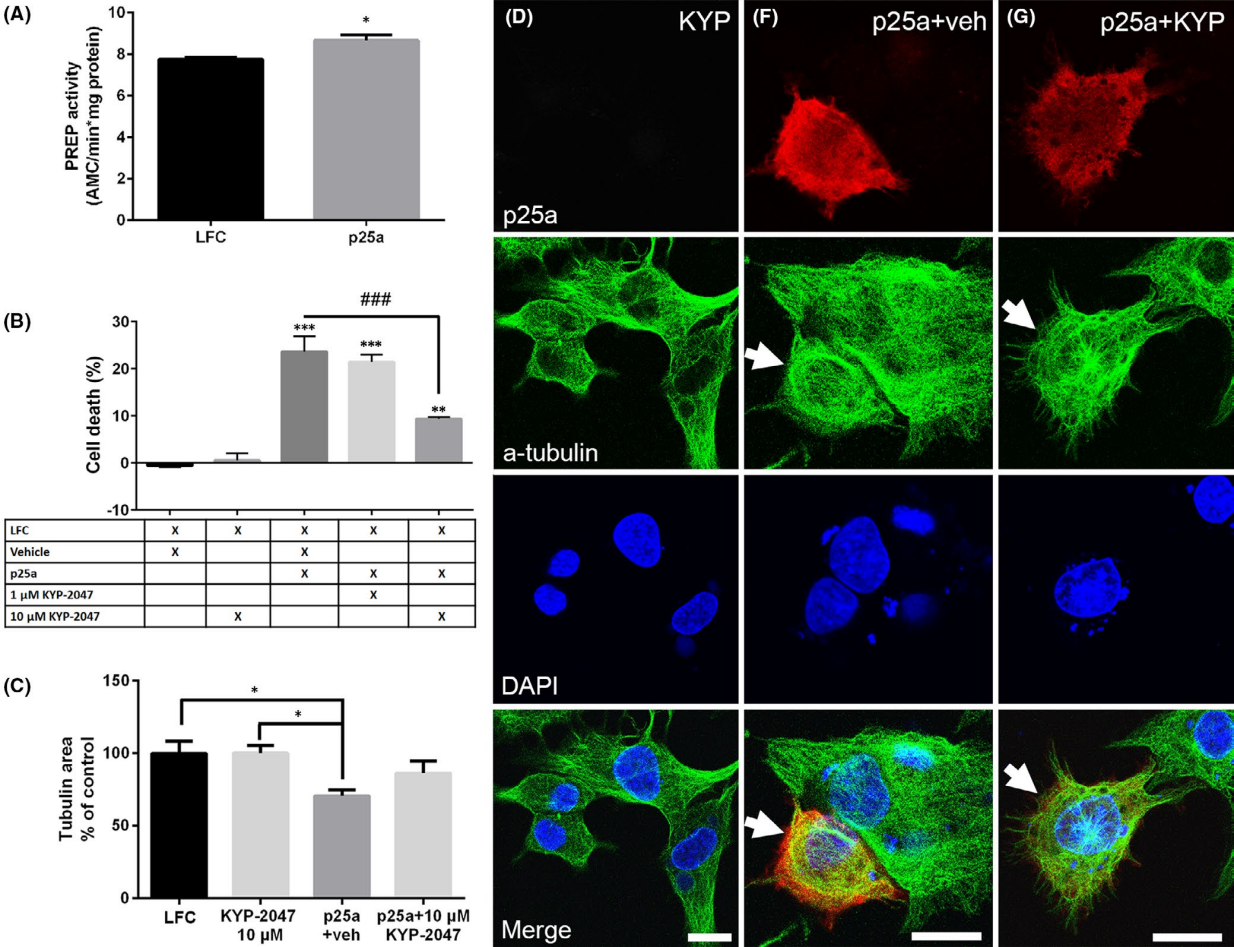
PREP activity increases after p25α transfection and 10 µM KYP‐2047 attenuates p25α toxicity. Transfection of OLN‐AS7 cells with p25α (p25a) caused small but significant increase in PREP activity compared to Lipofectamine control (LFC) (A). p25α transfection for 48 h also caused significant increase in cell death in the LDH assay compared to LFC control. However, 48 h PREP inhibition with 10 µM KYP‐2047 after p25α significantly reduced cellular death compared to p25α+vehicle (veh; 0.01% DMSO) (B). p25α transfection with vehicle treatment caused significant tubulin retraction compared to LFC or 10 µM KYP‐2047 alone while similar impact was not seen between LFC and p25α+10 µM KYP‐2047 (C). Representative immunofluorescence pictures showing the effect of p25α transfection (red) on α‐tubulin (a‐tubulin) network (green). 48 h treatment with 10 µM KYP‐2047 without p25α transfection had no effect on tubulin (D). p25α transfection and 48h vehicle treatment led to tubulin retraction (E) but the effect of p25α was reduced in cells treated for 48 h with 10 µM KYP‐2047 (F). Number of analysed cell/group in panel C is 30 (LFC); 25 (10 µM KYP‐2047); 27 (p25a+veh); 24 (p25a+10 µM KYP‐2047). DAPI was used as a nuclear marker, scale bar is 5 µM. Data are presented as mean ± SEM. *, *p* < 0.05, Student's *t*‐test (A). *, *p* < 0.05; ***, *p* < 0.001, 1‐way ANOVA with Tukey's post hoc test (B‐C)

Transfection of rat oligodendrocyte cell line stably overexpressing aSyn (OLN‐AS7 cells) with p25α caused a 11% increase in the PREP activity compared to empty Lipofectamine (LFC) transfection (Figure 1A; *t* = 3.338, *p* = 0.0289, Student's *t*‐test). p25α transfection also induced cellular death in the LDH cell viability assay 48 h after transfection (Figure 1B; 24% increase compared to LFC control; F_5,35_ = 29.32, *p *< 0.001, 1‐way ANOVA with Tukey's post‐test) but when OLN‐AS7 cells were treated simultaneously with 10 µM KYP‐2047, cell survival increased significantly (Figure 1B; F_5,35_ = 29.32, *p* < 0.001, 1‐way ANOVA with Tukey's post‐test). Based on cell viability studies, 10 µM KYP‐2047 was selected for further assays.

Translocation of p25α to the cytosol leads to loss of its normal function in myelination and this causes microtubule retraction contributing to cellular toxicity.^10^, ^23^ Similarly to previous studies, our ICC assay showed that transfected p25α caused significant decrease in the tubulin area in 48 h compared to LFC control or 10 µM KYP‐2047 (Figure 1C‐F; F_3,73_ = 1.358, *p* < 0.05, 1‐way ANOVA with Tukey's post‐test). When p25α transfection was combined with 10 µM KYP‐2047, the tubulin retraction was not significant compared to LFC but also not significantly different from p25α+vehicle (Figure 1C).

### 3.2 PREP inhibition reduces insoluble total and phosphorylated aSyn in OLN‐AS7 cells

**FIGURE 2:**
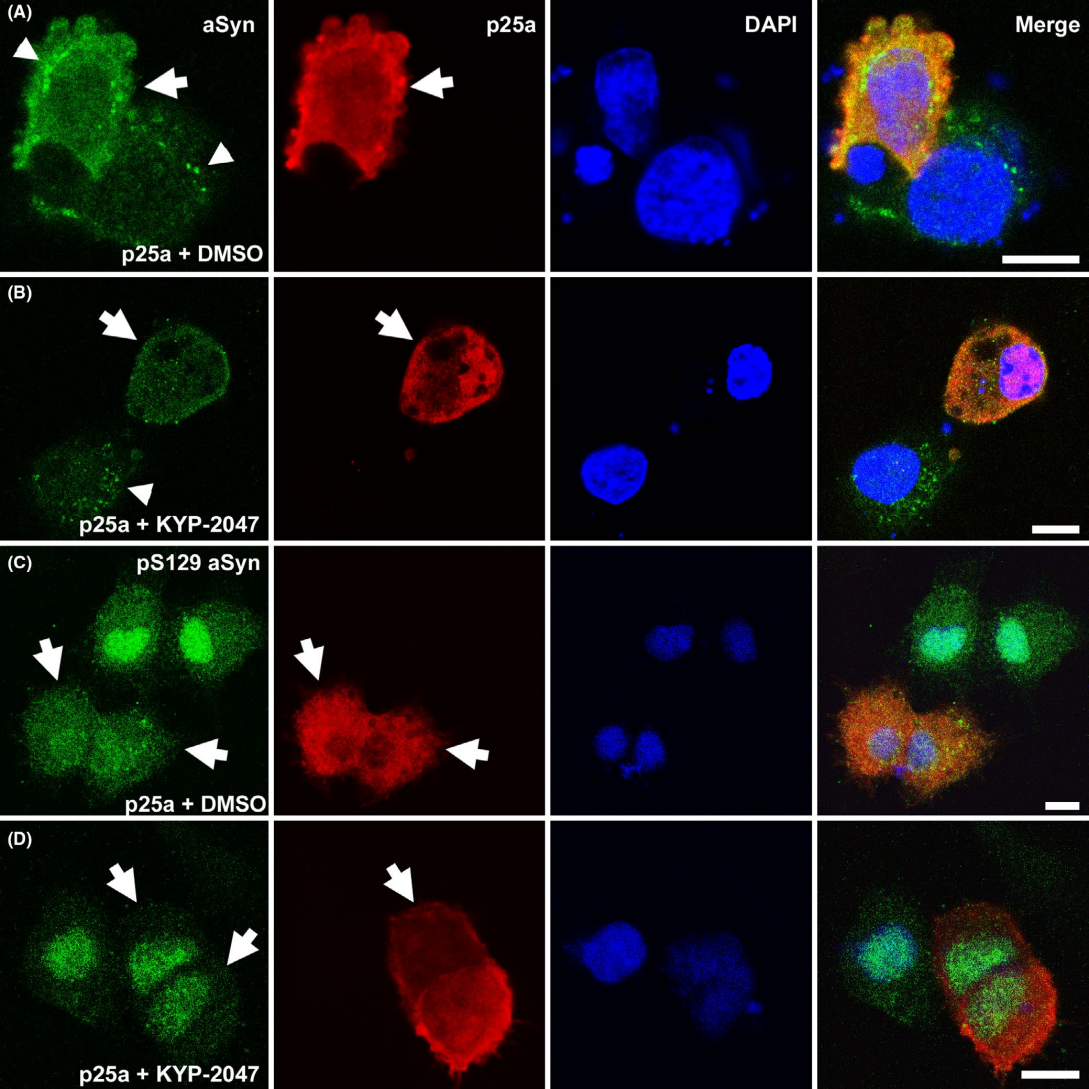
KYP‐2047 reduces cytosolic immunoreactivity in p25α‐transfected cells. In cells treated for 48 h with vehicle after p25α transfection (p25a+DMSO), p25α expressing cells (red; white arrow) showed intense cytosolic total aSyn immunostaining (A; green). When cells were incubated for 48 h with 10 µM KYP‐2047, cytosolic aSyn signal was lowered (B; p25a+KYP‐2047). OLN‐AS7 cells expressed aSyn puncta (white arrowheads) that were present both in p25α transfected and non‐transfected cells (cells without p25α expression (red colour)), and KYP‐2047 treatment seemed not to remove them from the cytosol (A‐B). Serine 129 phosphorylated aSyn (pS129 aSyn) showed strong nuclear staining but in p25α‐positive cells that were treated with DMSO for 48 h, the staining was also intense in the cytosol (C). 10 µM KYP‐2047 reduced cytosolic signal of pS129 aSyn (D). DAPI was used as a nuclear marker, scale bar is 5 µm

An earlier study with OLN‐AS7 cells showed that aSyn phosphorylation contributes to microtubule retraction and aSyn toxicity.^23^ PREP inhibition has reduced the number of aSyn oligomeric particles and particularly insoluble aggregates in our earlier studies,^17^, ^21^, ^26^ and therefore, we characterized if KYP‐2047 has an impact on aSyn phosphorylation and aggregation in OLN‐AS7 cells. We first tested 24 h p25α transfection but the WB analysis revealed the increase in aSyn and pS129 aSyn levels particularly in SDS fraction were mild (Figure S1). p25α toxicity has been shown to be the most evident at 48 h after p25α transfection in OLN‐AS7 cells,^23^ and therefore, we selected this time‐point for further studies. ICC showed that cells transfected with p25α had increased cytosolic aSyn immunoreactivity (Figure 2A). Interestingly, in p25α expressing cells, aSyn immunoreactivity was both diffuse and puncta‐like while in non‐transfected cells the staining pattern showed mostly puncta‐like structures (Figure 2A‐B). After transfection, 48 h treatment with KYP‐2047 reduced aSyn immunoreactivity in p25α‐positive cells but the puncta‐like staining remained in non‐transfected cells (Figure 2B). pS129 aSyn showed nuclear expression in OLN‐AS7 but in p25α transfected cells, more intense immunostaining was detected also in the cytosol (Figure 2C). 48 h KYP‐2047 incubation reduced cytosolic pS129 aSyn signal in p25α transfected cells (Figure 2D).

**FIGURE 3:**
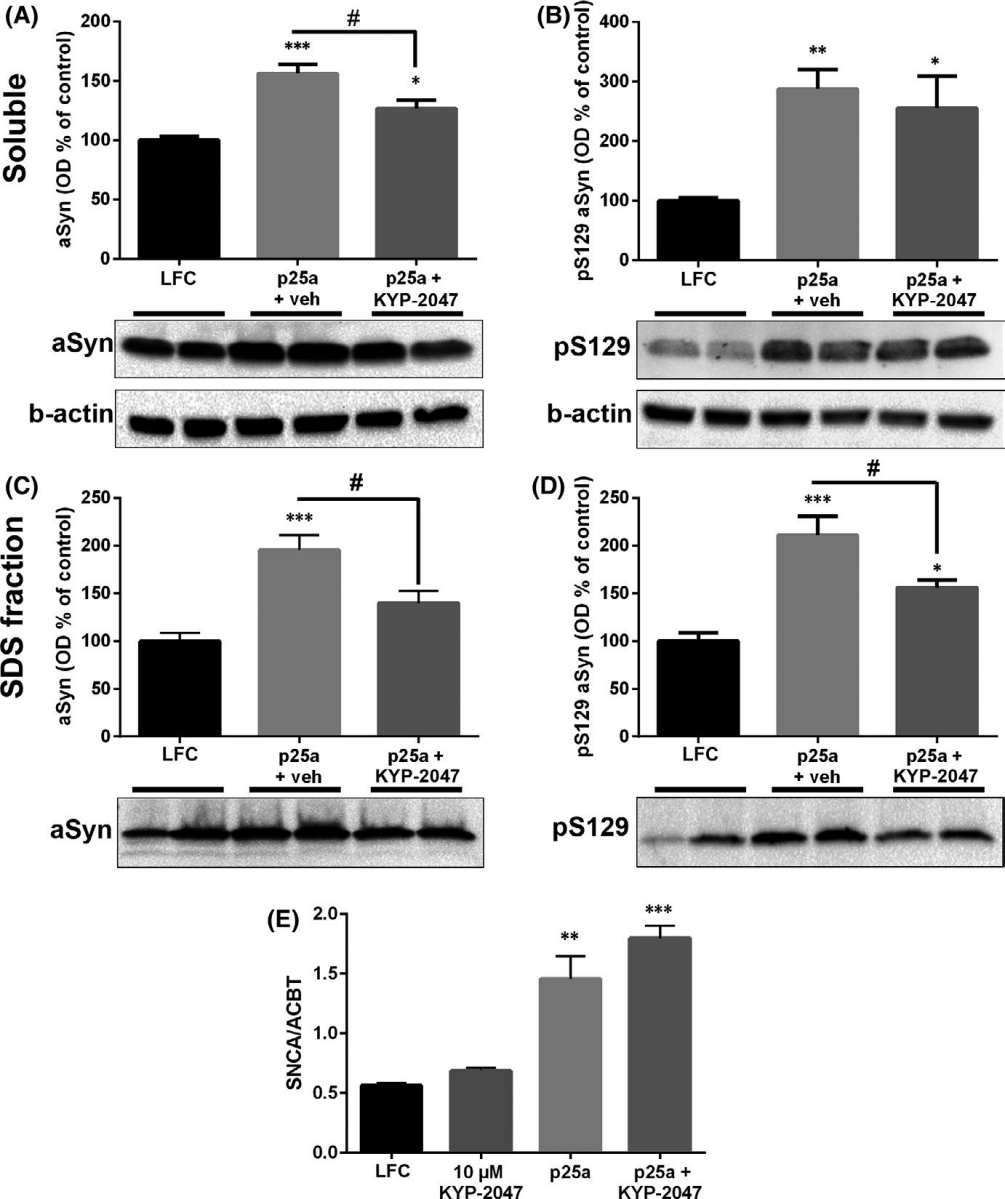
KYP‐2047 treatment reduces the levels of aSyn and phosphorylated aSyn in p25α transfected OLN‐AS7 cells. p25α transfection (p25α+veh; 0.01% DMSO as vehicle) increased the levels of total aSyn and Ser129 phosphorylated aSyn (pS129) in the soluble fraction (A‐B). 48 h treatment with 10 µM KYP‐2047 (p25α+KYP‐2047) significantly reduced total aSyn levels compared to vehicle treatment (p25α+veh) (A) but did not have an effect on soluble pS129 aSyn (B). In the SDS fraction, SDS‐soluble (monomeric) aSyn and pS129 aSyn were elevated after p25α transfection but 48 h treatment with KYP‐2047 significantly decreased the levels for both proteins (C‐D). p25α transfection elevated significantly the levels of aSyn mRNA (*Snca*) when correlated with the housekeeping gene β‐actin (*Acbt*; E). p25α transfection combined with 48 h KYP‐2047 treatment elevated *Snca* levels as well but this was not seen in protein levels (E). Data are presented as mean ± SEM. *, *p* < 0.05; **, *p* < 0.01; ***, *p* < 0.001, 1‐way ANOVA with Tukey's post hoc test (compared to vehicle). #, *p* < 0.05; 1‐way ANOVA with Tukey's post hoc test (p25α+veh vs. p25α+KYP‐2047). See figure S4 for uncut blots and for total protein loading control for insoluble fraction

After ICC, we confirmed the changes in protein levels in the soluble faction and the SDS fraction by WB. WB analysis revealed that at the 48 h time‐point total and pS129 aSyn were significantly elevated in the soluble fraction of p25α transfected cells (Figure 3A‐B; total aSyn, F_2,15_ = 19.45, *p* < 0.001 LFC vs. p25α+vehicle, *p* < 0.05 LFC vs. p25α+KYP‐2047; pS129 aSyn, F_2,15_ = 7.25, *p* < 0.01 LFC vs. p25α+vehicle, *p* < 0.05 p25α+KYP‐2047, 1‐way ANOVA with Tukey's post hoc test). KYP‐2047 decreased total aSyn levels compared to vehicle treatment but not the pS129 aSyn levels (Figure 3A‐B; *p* < 0.05, p25α+vehicle vs. p25α+KYP‐2047, 1‐way ANOVA with Tukey's post‐test). Both analysed aSyn forms were also increased in SDS fraction in p25α transfected groups (Figure 3A‐D; total aSyn, F_2,21_ = 14.51, *p* < 0.001 LFC vs. p25α+vehicle; pS129 aSyn; F_2,21_ = 16.61, *p* < 0.001 LFC vs. p25α+vehicle, *p* < 0.05 LFC vs. p25α+KYP‐2047, 1‐way ANOVA with Tukey's post hoc test). 48 h treatment with 10 µM KYP‐2047 significantly decreased both total and pS129 aSyn compared to vehicle treatment (Figure 3C‐D; *p* < 0.05, 1‐way ANOVA with Tukey's post hoc test). Although clear aSyn and pS129 aSyn signal were detected in the SDS fraction, pointing to aggregation and formation of non‐soluble aSyn forms, mostly monomeric aSyn was seen in the SDS fraction (Figure 3A‐D; see full membranes and total protein for insoluble fraction in Figure S4). The ratios between pS129 aSyn and aSyn remained unchanged in soluble and SDS fraction but the ratio between insoluble/soluble pS129 aSyn was significantly increased in p25α+vehicle group (Figure S2; F_2,17_ = 6.784, *p* < 0.05 LFC vs. p25α+vehicle, 1‐way ANOVA with Tukey's post hoc test).

Since the levels of aSyn were significantly elevated after p25α transfection, we measured if mRNA levels of aSyn (*Snca*) had changed. Interestingly, p25α transfection alone increased the levels of *Snca* mRNA (Figure 3E; F_3,8_ = 28.59, *p* < 0.001 NC vs. p25α; *p* < 0.0001 p25α vs. p25α+KYP‐2047; 1‐way ANOVA with Tukey's post‐test) that explains changes in protein levels and also different staining pattern in ICC (Figure 2). KYP‐2047 did not have effect on *Snca* mRNA levels, similar to an earlier study,^26^ and KYP‐2047 did not alter p25α levels in OLN‐AS7 cells (Figure S3).

### 3.3 PREP inhibition induces autophagy and re‐activates protein phosphatase 2A (PP2A) in OLN‐AS7 cells

**FIGURE 4:**
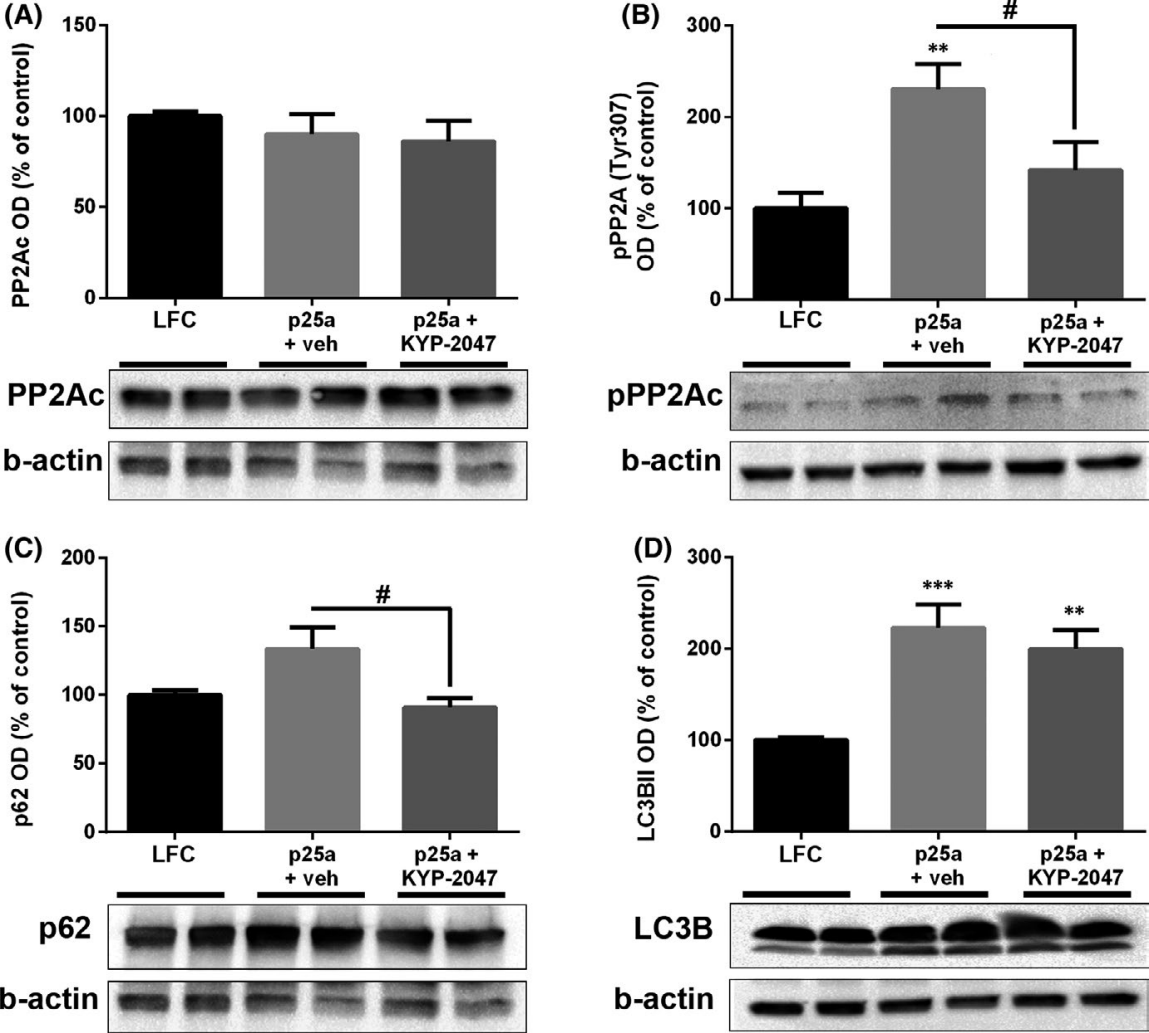
PREP inhibition reduces PP2A phosphorylation and activates autophagy in p25α transfected OLN‐AS7 cells. p25α transfection did not alter the levels of catalytic subunit of PP2A (A; PP2Ac) but Tyr307 phosphorylated PP2Ac (B; pPP2Ac) was significantly elevated after transfection and vehicle treatment (B). 10 µM KYP‐2047 significantly reduced pPP2Ac levels compared to p25α+vehicle but did not change the levels of PP2Ac (A‐B). The protein accumulation marker, p62, was significantly decreased in p25α transfected and KYP‐2047 treated cells compared to vehicle treatment (C). Autophagosome marker LC3BII was elevated in p25α transfected cells and remained elevated after 10 µM KYP‐2047 treatment (D). Data are presented as mean ± SEM. *, *p* < 0.05; **, *p* < 0.01; ***, *p* < 0.001, 1‐way ANOVA with Tukey's post hoc test (compared to vehicle). #, *p* < 0.05; 1‐way ANOVA with Tukey's post hoc test (p25α+veh vs. p25α+KYP‐2047). See figure S5for uncut blots. Representative blots in Figure 4A and C are from the same membrane and therefore share the beta‐actin loading control

Our previous results show that PREP inhibition can activate autophagy to reduce aSyn aggregates,^16^, ^17^, ^27^ and we recently showed that PREP inhibition induces autophagy by activating PP2A.^25^ We studied the levels of the total catalytic subunit of PP2A (PP2Ac) and inactive Tyr307 phosphorylated PP2Ac (pPP2A; antibody specific for inactive PP2A verified in^25^) by WB after p25α transfection and 48 h treatment by vehicle or KYP‐2047. This revealed that p25α transfection significantly elevated the levels of pPP2A (Figure 4B; F_2,15_ = 6.619, *p* < 0.01 LFC vs. p25α+vehicle, 1‐way ANOVA with Tukey's post hoc test), indicating PP2A inactivation, while treatment with 10 µM KYP‐2047 restored pPP2A levels close to control level (Figure 4B; F_2,15_ = 6.619, *p* < 0.01 p25α+vehicle vs. p25α+KYP‐2047, 1‐way ANOVA with Tukey's post hoc test).

When we detected the autophagy markers–protein accumulation marker p62 and autophagosome marker LC3BII–in OLN‐AS7 cells after p25α transfection, LC3BII was significantly elevated both in vehicle and KYP‐2047 treated cells (Figure 4D; LC3BII; F_2,20_ = 12.80, *p* < 0.001 LFC vs. p25α+vehicle; *p* < 0.01 LFC vs. p25α+KYP‐2047, 1‐way ANOVA with Tukey's post hoc test). p62 was elevated in p25α+vehicle group but this was not significant (Figure 4C). However, 10 µM KYP‐2047 significantly decreased the p62 levels compared to vehicle‐treated cells (Figure 4C; F_2,20_ = 5.464, *p* < 0.05 p25α+vehicle vs. p25α+KYP‐2047, 1‐way ANOVA with Tukey's post hoc test). Since p62 was not decreased in p25α+vehicle group and LC3BII levels were also increased, this suggests decreased degradation of autophagosomes and reduced autophagic flux.^28^ In line with decreased aSyn levels in the SDS fraction, 48 h KYP‐2047 incubation decreased the levels of p62 that in combination with elevated LC3BII levels indicates increased autophagic flux (Figure 4C‐D; both detected from soluble fraction). This also explains why KYP‐2047 decreased aSyn levels in ICC and WB although the mRNA expression was not changed.

### 3.4 p25α enhances aSyn accumulation and PP2Ac phosphorylation in human oligodendrocyte cells

**FIGURE 5:**
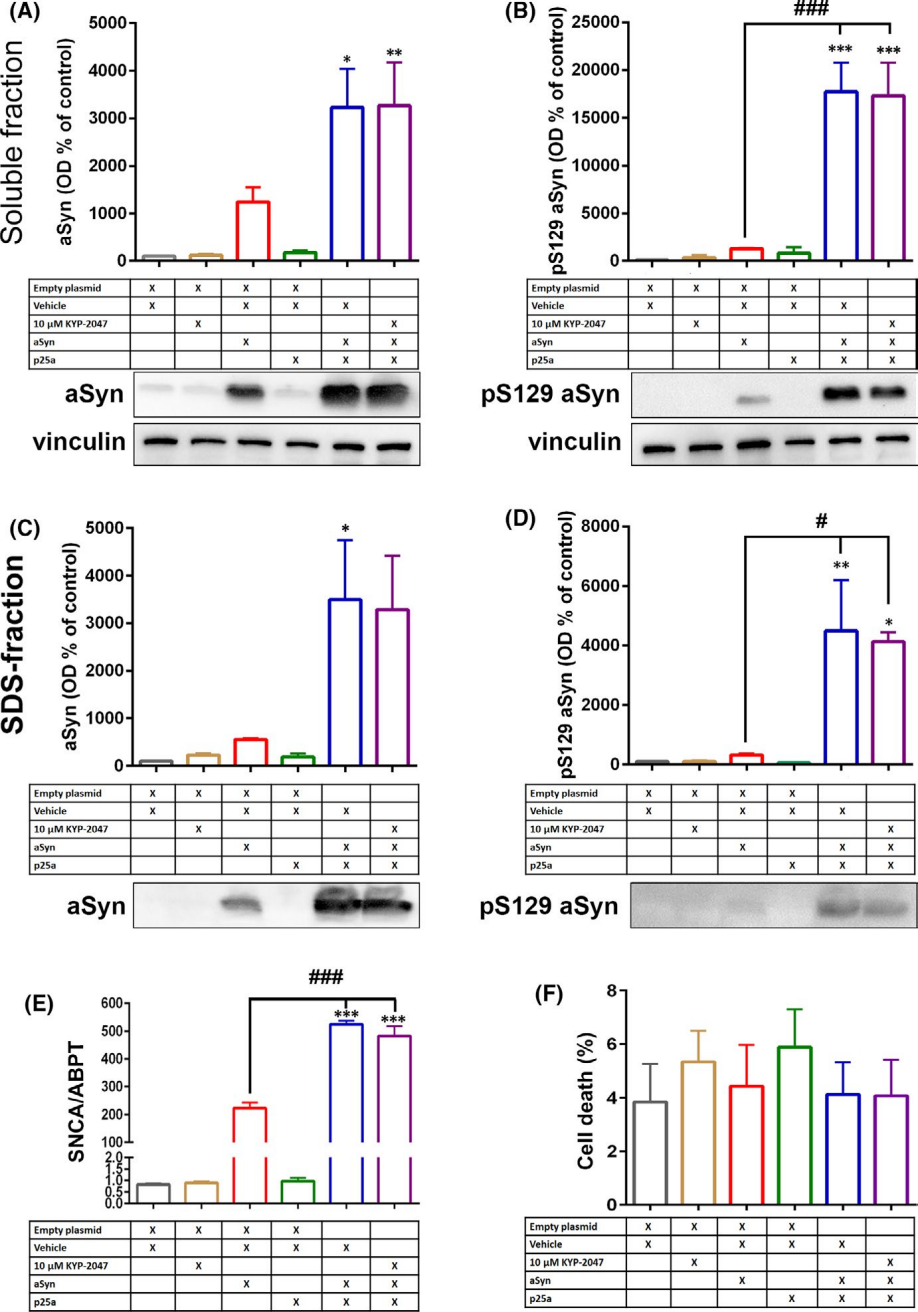
p25α transfection elevates aSyn levels and phosphorylation in MO3.13 cells. p25α co‐transfection with aSyn elevated aSyn levels both in soluble and SDS fraction (A, C). After 48h co‐transfection, pS129 aSyn was increased significantly compared to empty control and aSyn+empty in both fractions (B, D). This was in correlation with mRNA levels where similar increase in aSyn mRNA expression was seen when co‐transfected with p25α (E; SNCA). However, no changes in cell viability were seen by any transfections or treatments (F), and 10 µM KYP‐2047 had no effect on aSyn levels or aSyn mRNA. Data are presented as mean ± SEM. *p* < 0.05; **, *p* < 0.01; ***, *p* < 0.001, 1‐way ANOVA with Tukey's post hoc test (compared to empty plasmid). #, *p* < 0.05; ###, *p* < 0.001 1‐way ANOVA with Tukey's post hoc test (aSyn+empty+veh vs. p25α+aSyn+veh or p25α+aSyn+KYP‐2047). See figure S6 for uncut blots and for total protein loading control for insoluble fraction. Representative blots in Figures 5A and 6C are from the same membrane and therefore share the vinculin loading control

We wanted to verify the main results seen in OLN‐AS7 cells with human oligodendrocyte cell line MO3.13. Cells were transfected with p25α or aSyn +/− p25α, and treated with 10 µM KYP‐2047 for 48 h. Co‐transfection of p25α and aSyn caused significant increase in soluble aSyn compared to empty plasmid (Figure 5A; F_5,12_ = 8.842, *p* < 0.05 empty vs. p25α+vehicle; *p* < 0.01 empty vs. p25α+KYP‐2047, 1‐way ANOVA with Tukey's post hoc test). Similar to aSyn, pS129 aSyn was also significantly elevated compared to empty transfection (Figure 5B; F_5,12_ = 20.98, *p* < 0.001 empty vs. p25α+vehicle; *p* < 0.001 empty vs. p25α+KYP‐2047, 1‐way ANOVA with Tukey's post hoc test) but combination of p25α and aSyn also significantly elevated the levels of pS129 aSyn compared to aSyn alone (Figure 5B; F_5,12_ = 20.98, *p* < 0.001 aSyn+empty vs. p25α+vehicle; *p* < 0.001 aSyn+empty vs. p25α+KYP‐2047, 1‐way ANOVA with Tukey's post hoc). Changes in aSyn levels can be explained by positive impact of p25α on aSyn mRNA expression similar to OLN‐AS7 cells (Figure 5E; F_4,12_ = 1.286, *p* < 0.001 p25α+vehicle vs. p25α+aSyn and p25α+aSyn+KYP‐2047, 1‐way ANOVA with Tukey's post hoc) but significant increase in pS129 aSyn by p25α co‐transfection points to p25α catalysed aSyn phosphorylation. In the insoluble fraction, p25α elevated similarly insoluble aSyn but this was not statistically different from aSyn alone (Figure 5C; F_5,12_ = 5.481, *p* < 0.05 empty vs. p25α+vehicle). However, also insoluble pS129 aSyn was significantly elevated compared to aSyn alone (Figure 5D; F_5,12_ = 9.115, *p* < 0.01 empty vs. p25α+vehicle, *p* < 0.05 empty vs. p25α+KYP‐2047; *p* < 0.05 aSyn+empty vs. p25α+vehicle and p25α+KYP‐2047, 1‐way ANOVA with Tukey's post hoc). However, 10 µM KYP‐2047 had no impact on any aSyn forms in MO3.13 cells (Figure 5A‐D). Additionally, aSyn or aSyn and p25α co‐transfection had no effect on MO3.13 cell viability (Figure 5F).

**FIGURE 6:**
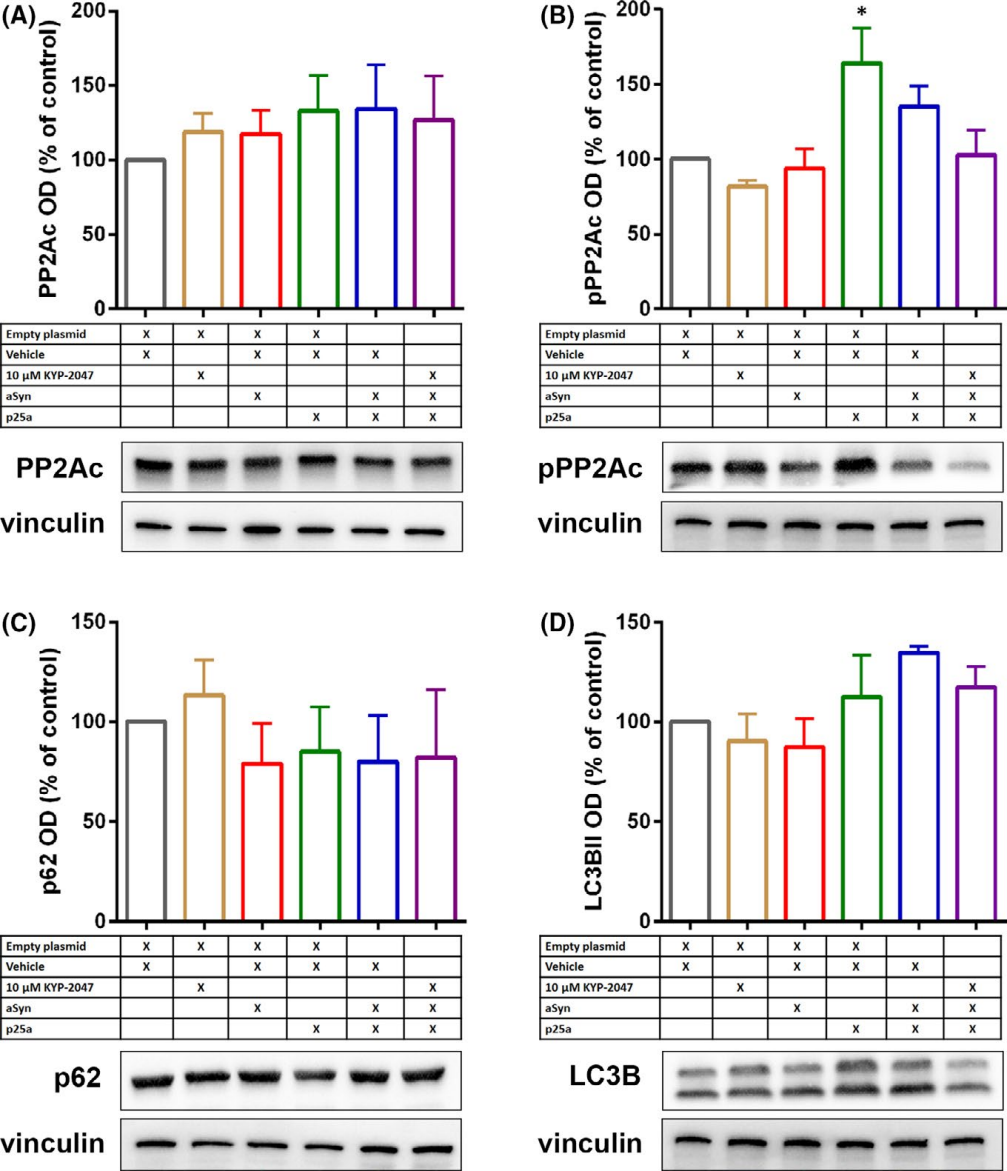
p25α transfection increases PP2A phosphorylation in MO3.13 cells. Similar to OLN‐AS7 cells, p25α, aSyn or p25α+aSyn transfection did not alter the levels of catalytic subunit of PP2A (A; PP2Ac). However, Tyr307 phosphorylated PP2Ac (B; pPP2Ac) was significantly increased by p25α transfection but not with other combinations. 10 µM KYP‐2047 reduced pPP2Ac levels but not significantly (B). p62 or autophagosome marker LC3BII were not altered by p25α, aSyn or their combination in MO3.13 cells (C‐D). Data are presented as mean ± SEM. *, *p* < 0.05; empty+veh vs. p25α+veh, 1‐way ANOVA with Tukey's post hoc test. See figure S7 for uncut blots. Representative blots in Figure 6B and D are from the same membrane and therefore share the vinculin loading control

We further characterized the levels of PP2Ac, pPP2Ac and autophagy markers LC3B and p62 from soluble fractions of MO3.13 cells. Similar to OLN‐AS7 cells, total levels of PP2Ac were not altered (Figure 6A) but p25α transfection alone significantly elevated pPP2Ac levels (Figure 6B; F_5,18_ = 4.700, *p* < 0.05 empty vs. p25α+empty, 1‐way ANOVA with Tukey's post‐test). Interestingly, co‐transfection of p25α and aSyn did not have significant effect on pPP2Ac and although KYP‐2047 decreased the inactive form of PP2Ac, this was not significant (Figure 6B). Unlike in OLN‐AS7 cells, autophagy markers were not altered by p25α and aSyn (Figure 6C‐D).

## 4 DISCUSSION

MSA is a devastating neurodegenerative disease that lacks disease‐modifying therapy. In this study, we have shown that KYP‐2047, a PREP inhibitor that has shown beneficial effects in several aSyn‐based PD models, protects cells from aSyn toxicity in a cellular model of MSA. Our results demonstrate that in OLN‐AS7 cells, KYP‐2047 induces autophagy by re‐activating PP2A and this leads to decreased levels of aSyn aggregates and elevated cell viability.

Alterations in PREP activity have been reported in several neurodegenerative diseases, including Parkinson's and Alzheimer's diseases,^29^, ^30^ but there are no reports about PREP in MSA. PREP activity has been reported in rat oligodendrocytes,^31^ but the activity was lower than in neurons or astrocytes. However, in the current study, the PREP activity measured from OLN‐AS7 cells was remarkably high when compared, for example to SH‐SY5Y neuronal cell culture^26^ and p25α transfection elevated PREP activity. It has been shown that PREP activity and levels are increased in astrocytes and microglial cells when they are activated, suggesting that PREP is inducible in glial cell activation.^29^, ^32^ Therefore, it could be possible that PREP is also induced in MSA after p25α translocation but this requires further studies by using MSA patient material.

Our results revealed that p25α transfection in OLN‐AS7 cells caused increased phosphorylation of PP2A at Tyr307 that is indicative for PP2A inactivation.^33^ Interestingly, our recent study showed that PREP negatively regulates PP2A phosphatase functions via protein‐protein interactions.^25^ Elevated PREP activity after p25α transfection could contribute to reduced PP2A levels but we cannot exclude the impact of p25α itself. Lowered PP2A activity has been connected particularly to Alzheimer's disease and tauopathies since PP2A is the most important dephosphorylating phosphatase for Tau.^34^ Decreased PP2A levels and activity have been measured in synucleinopathies as well,^35^, ^36^ and PP2A can dephosphorylate aSyn at Ser129 thus stabilizing it (see below).^37^ Elevated aSyn levels are shown to increase PP2A activity *in vitro* ^38^ but aggregated aSyn decreases PP2A activity.^39^ PP2A regulates, for example autophagy (see below), glutamate receptor recycling and protein stability via dephosphorylation, and decreased PP2A activity can result in several forms of cellular damage in neurodegenerative diseases.^40^, ^41^ There are no reports about PP2A levels in MSA, or a connection between p25α and PP2A, but our finding suggests that this should be further studied and that PP2A inactivation could contribute to MSA as well.

p25α transfection in both cell lines elevated the levels of aSyn and pS129 aSyn both in soluble and insoluble fractions that may well cause cellular toxicity.^10^, ^23^ Interestingly, p25α transfection also increased the levels of aSyn mRNA in both cell lines, suggesting that translocation of p25α could induce aSyn expression and this way elevate aSyn levels in oligodendrocytes. However, aSyn mRNA has not been reported to be significantly elevated in oligodendrocytes of MSA patients,^9^ and our finding requires further verification. Our data shows that KYP‐2047 treatment reduced the levels of insoluble pS129 aSyn but had no effect on the levels of soluble pS129 aSyn in OLN‐AS7 cells. However, Similar impact by KYP‐2047 was not seen in MO3.13 cells. This could be due the enormous aSyn overexpression that is significantly higher than, for example in stably aSyn overexpressing SH‐SY5Y cells where PREP inhibitors have shown good effects.^26^ Additionally, the aSyn plasmid used in the study has a V5‐tag in its C‐terminus.^17^ The results by Savolainen et al. (2015)^15^ showed that truncation of C‐terminus disturbs the interaction between PREP and aSyn, and it is possible that tagged aSyn is not optimal for PREP interaction, and therefore, the impact of PREP inhibitor is also altered. This view is supported also by an earlier study by Svarcbahs et al. (2018),^16^ where the same plasmid was used but the deletion of PREP did not have significant effect on aSyn levels although PREP deleted HEK‐293 cells had significantly accelerated autophagic flux.

Phosphorylation of aSyn has been under intensive studies since normally only a small portion of brain aSyn is phosphorylated but in synucleinopathies the ratio of pS129 aSyn increases substantially.^5^ However, it is still under debate how this post‐translational modification is related to synucleinopathies and aSyn toxicity. aSyn phosphorylation at serine 129 may change its membrane binding and interaction partners, and it also increased nuclear localization of aSyn that may interfere with gene functions^42^ but toxicity of pS129 aSyn *in vivo* has remained controversial (for review, see^43^). On the other hand, aSyn phosphorylation at serine 129 increases its turnover in cells, and it has been suggested that Ser129 phosphorylation targets aSyn for proteasomal degradation.^44^ Therefore, it is possible that when KYP‐2047 induced autophagy, this reduced proteasomal activity in the cells (for review about the interplay between proteasomal and autophagy systems, see^45^) as we have reported in PREP knock‐out HEK‐293 cells,^16^ and caused the increase in soluble pS129 aSyn.

Monomeric aSyn is degraded via proteasomes and chaperone‐mediated autophagy but these systems are easily damaged by aSyn aggregates, leading to elevated aSyn concentrations and aggregation.^46^, ^47^ Macroautophagy (autophagy) is able to degrade aSyn oligomers and larger aggregates,^48^ and the activation of autophagy has been suggested as a possible therapy against synucleinopathies and other neurodegenerative diseases (for reviews, see^6^, ^49^). Impaired autophagy has been reported in MSA patient samples and patient‐derived induced pluripotent stem cells,^50^, ^51^ and it may well contribute to oligodendroglial aSyn accumulation and toxicity. We also showed here that in OLN‐AS7 cells, p25α transfection caused intracellular accumulation of autophagosomes and aggregation markers, indicating reduced autophagic flux. This is in line with a previous study, where p25α and aSyn together interfered with autophagosome‐lysosome interaction, and p25α alone impaired autophagosome formation.^52^ Although aSyn and p25α co‐transfection significantly elevated aSyn phosphorylation both in soluble and insoluble fractions, this did not cause any changes in autophagy markers in MO3.13 cells. aSyn and p25α overexpression also did not have effect on cell viability of MO3.13 cells, and this emphasizes the importance of normal autophagy for cellular toxicity of aSyn. It is possible that p25α‐induced PP2A inactivation is behind reduced autophagosome formation since PP2A regulates autophagy via mTOR and beclin1 pathways that are responsible for autophagy initiation and autophagosome formation, respectively.^53^, ^54^ Autophagy activators attenuated symptoms in a mouse model of MSA,^14^, ^55^ and our results with KYP‐2047 indicate that autophagy activation reduces p25α transfection induced aSyn toxicity in OLN‐AS7 cells by removing particularly insoluble aSyn species.

Taken together, our data suggest that p25α transfection to cellular models of MSA induces not only aSyn phosphorylation and aggregation but also PP2A inactivation. Additionally, our results suggest that autophagy impairment is important step for aSyn‐induced toxicity in cellular models of MSA. In OLN‐AS7 cells, PREP inhibition after p25α transfection re‐activated PP2A, reduced both total and pSer129 aSyn in the insoluble fraction by inducing autophagy and attenuated the toxicity of aSyn in in OLN‐AS7 cellular model of MSA. Our results suggest that PREP inhibition could be a possible disease‐modifying therapy that is applicable for several synucleinopathies.

## CONFLICTS OF INTEREST

The authors confirm that there are no conflicts of interest.

## AUTHOR CONTRIBUTIONS

**Hengjing Cui:** Formal analysis (equal); Investigation (equal); Methodology (equal); Validation (equal); Writing‐original draft (equal). **Tommi Kilpeläinen:** Conceptualization (equal); Formal analysis (equal); Funding acquisition (equal); Investigation (equal); Writing‐original draft (equal). **Lydia Zouzoula:** Investigation (equal); Methodology (equal); Writing‐original draft (equal). **Samuli Auno:** Conceptualization (equal); Formal analysis (equal); Funding acquisition (equal); Investigation (equal); Writing‐original draft (equal). **Kalevi Trontti:** Investigation (supporting); Methodology (supporting); Writing‐review & editing (supporting). **Sampo Kurvonen:** Investigation (supporting); Methodology (supporting); Writing‐review & editing (supporting). **Susanna Norrbacka:** Formal analysis (supporting); Investigation (supporting); Methodology (supporting). **Iiris Hovatta:** Project administration (supporting); Resources (supporting); Supervision (supporting); Writing‐review & editing (supporting). **Poul Henning Jensen:** Resources (supporting); Writing‐review & editing (supporting). **Timo T Myöhänen:** Funding acquisition (lead); Project administration (lead); Supervision (lead); Visualization (lead); Writing‐review & editing (lead).

## Supporting information

Supplementary Material

## Data Availability

All data generated or analysed during this study are included in this published article. Raw values are available from the corresponding author on reasonable request.
