# Supplementary material for: Prolyl oligopeptidase inhibition reduces alpha‐synuclein aggregation in a cellular model of multiple system atrophy
Source: J Cell Mol Med. 2021 Sep 5;25(20):9634–46. doi: 10.1111/jcmm.16910 (PMC8505845; doi:10.1111/jcmm.16910)
Supplement: Supplementary file 1 — Supplementary Material [file JCMM-25-9634-s001.pdf]

Prolyl oligopeptidase inhibition reduces alpha-synuclein aggregation in a cellular model of multiple system atrophy

Cui H[1], Kilpeläinen T[1], Zouzoula L[1], Auno S[1], Trontti K [2,3,4], Kurvonen S[1], Norrbacka S[1], Hovatta I [2,3,4], Jensen PH[5], Myöhänen TT[1,6,7#]

[1] Division of Pharmacology and Pharmacotherapy/Drug Research Program, University of Helsinki, Finland

[2] SleepWell Research Program, Faculty of Medicine, University of Helsinki, Finland.

[3] Department of Psychology and Logopedics, University of Helsinki, Finland.

[4] Neuroscience Center, University of Helsinki, Finland.

[5] Department of Biomedicine & DANDRITE, Aarhus University, Denmark

[6] Integrative Physiology and Pharmacology Unit, Institute of Biomedicine, University of Turku, Finland

[7] School of Pharmacy, University of Eastern Finland, Finland

# corresponding author:

Dr. Timo T. Myöhänen

Division of Pharmacology and Pharmacotherapy / Drug Research Programme

Faculty of Pharmacy

Viikinkaari 5E (PO Box 56)

00014 University of Helsinki

FINLAND

email: timo.myohanen@helsinki.fi

tel. +358-50-5641426

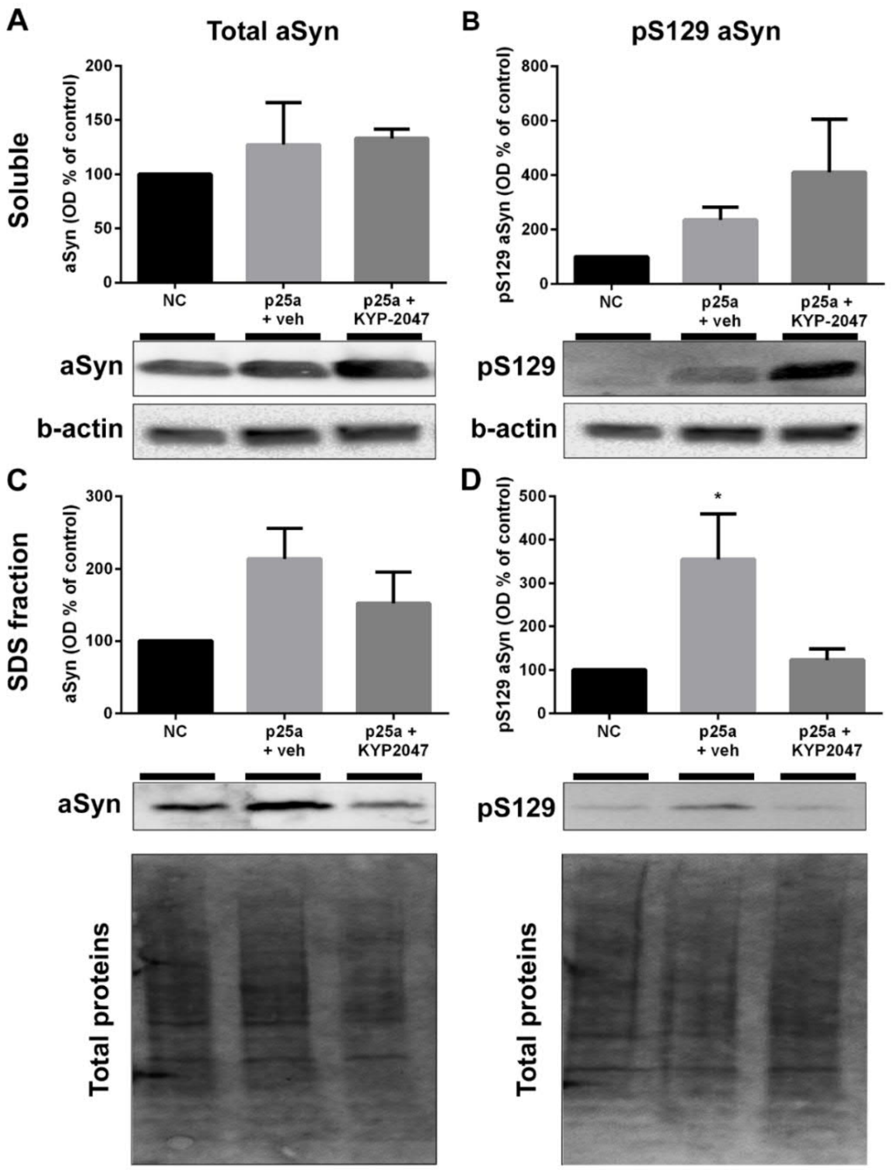

Figure S1. Effect of PREP inhibition on aSyn and pS129 aSyn aggregation after 24 h incubation. OLN-AS7 cells transfected with p25α showed elevated soluble total and serine 129 phosphorylated aSyn (pS129 aSyn) but this was not significant (A-B). In the SDS-fraction, aSyn and pS129 aSyn were elevated but only the phosphorylated form was significantly increased from control (C-D). 24 h incubation with 10 µM KYP-2047 reduced the levels of pS129 aSyn close to control levels (NC, negative control). Data is presented as mean ± SEM. *, $p<0.05$, 1-way ANOVA with Tukey's post test (D). See supplementary figure S8 for uncut blots.

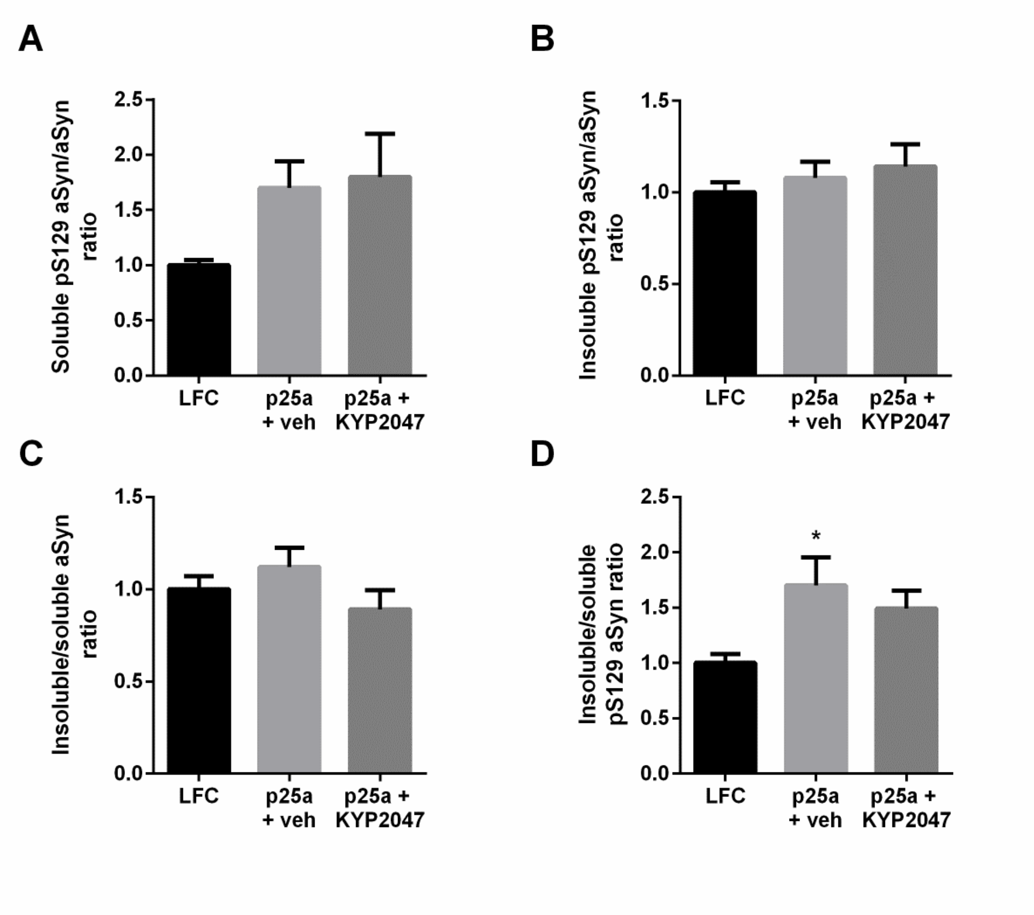

Figure S2. The effect of p25a transfection on aSyn ratios in OLN-AS7 cells. p25a did not alter the ratios between aSyn and pS129 aSyn (A-C) but significantly elevated the ratio between soluble and insoluble pS129 aSyn (D; $F_{2,17} = 6.784$, $p<0.05$ LFC vs. p25a+vehicle; 1-way ANOVA with Tukey's post-test).

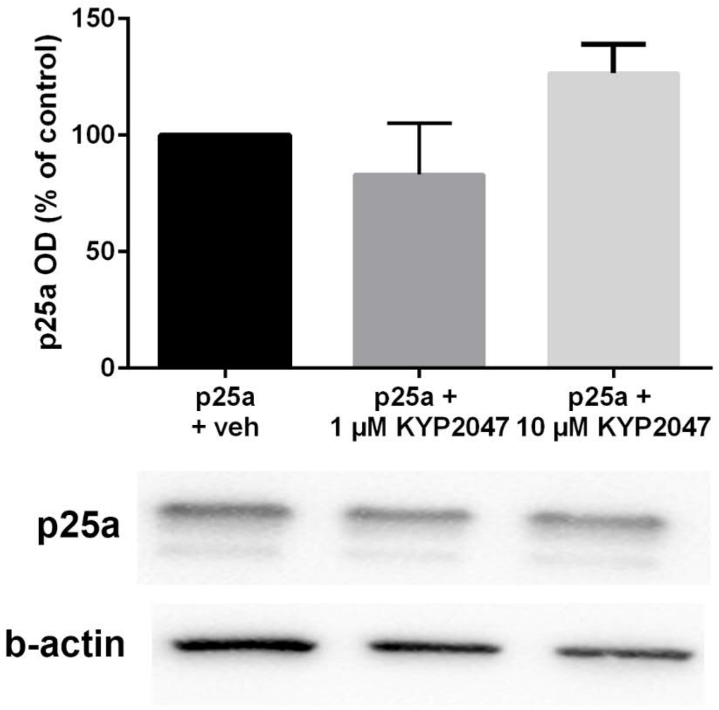

Figure S3. PREP inhibition has no effect on p25a levels after 48 h incubation. See Suppelementary Fig. S9 for uncut blots.

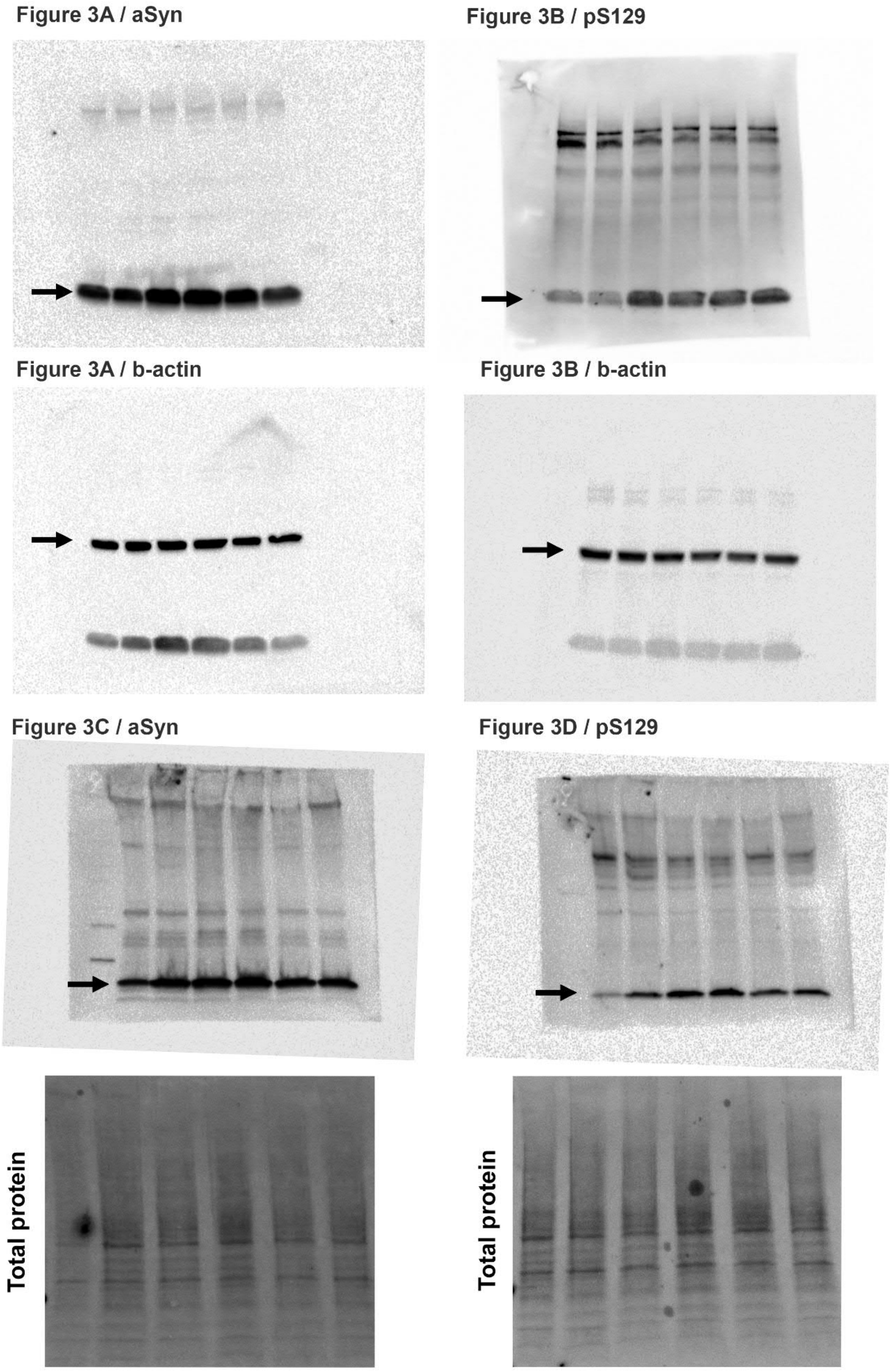

Figure S4. Uncut blots for Figure 3. Arrowhead marks the presented and analyzed band.

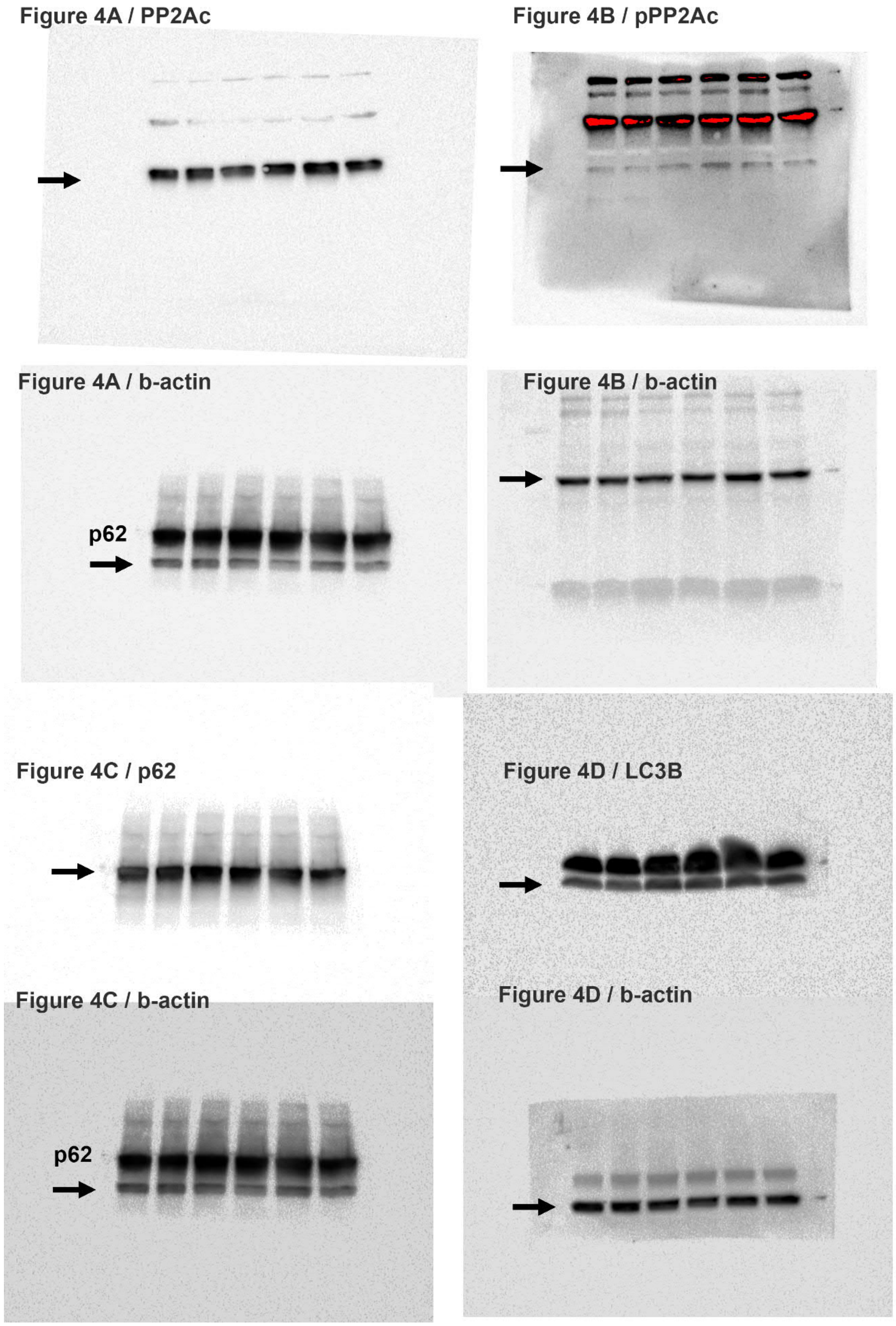

Figure S5. Uncut blots for Figure 4. Arrowhead marks the presented and analyzed band.

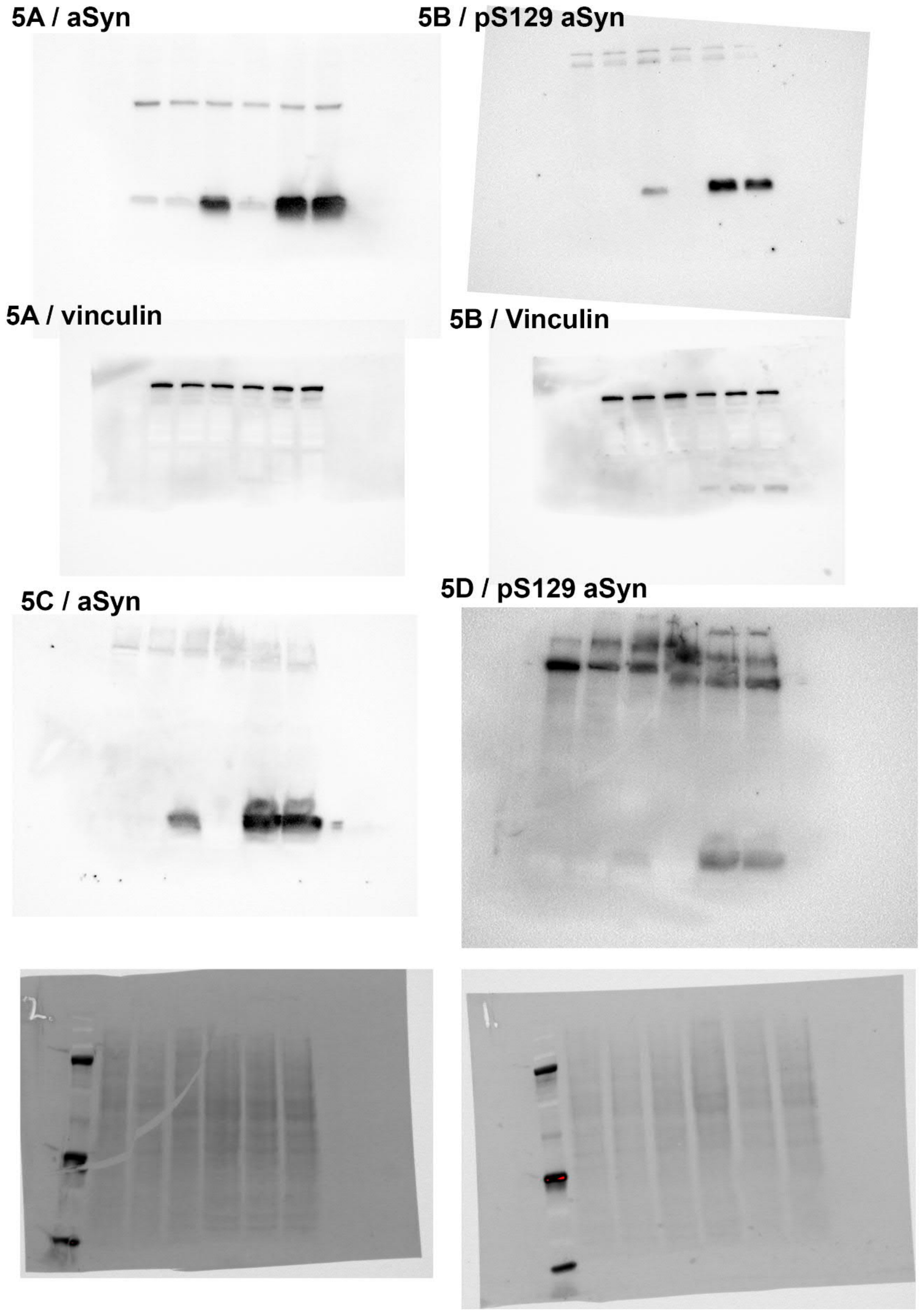

Figure S6. Uncut blots for Figure 5.

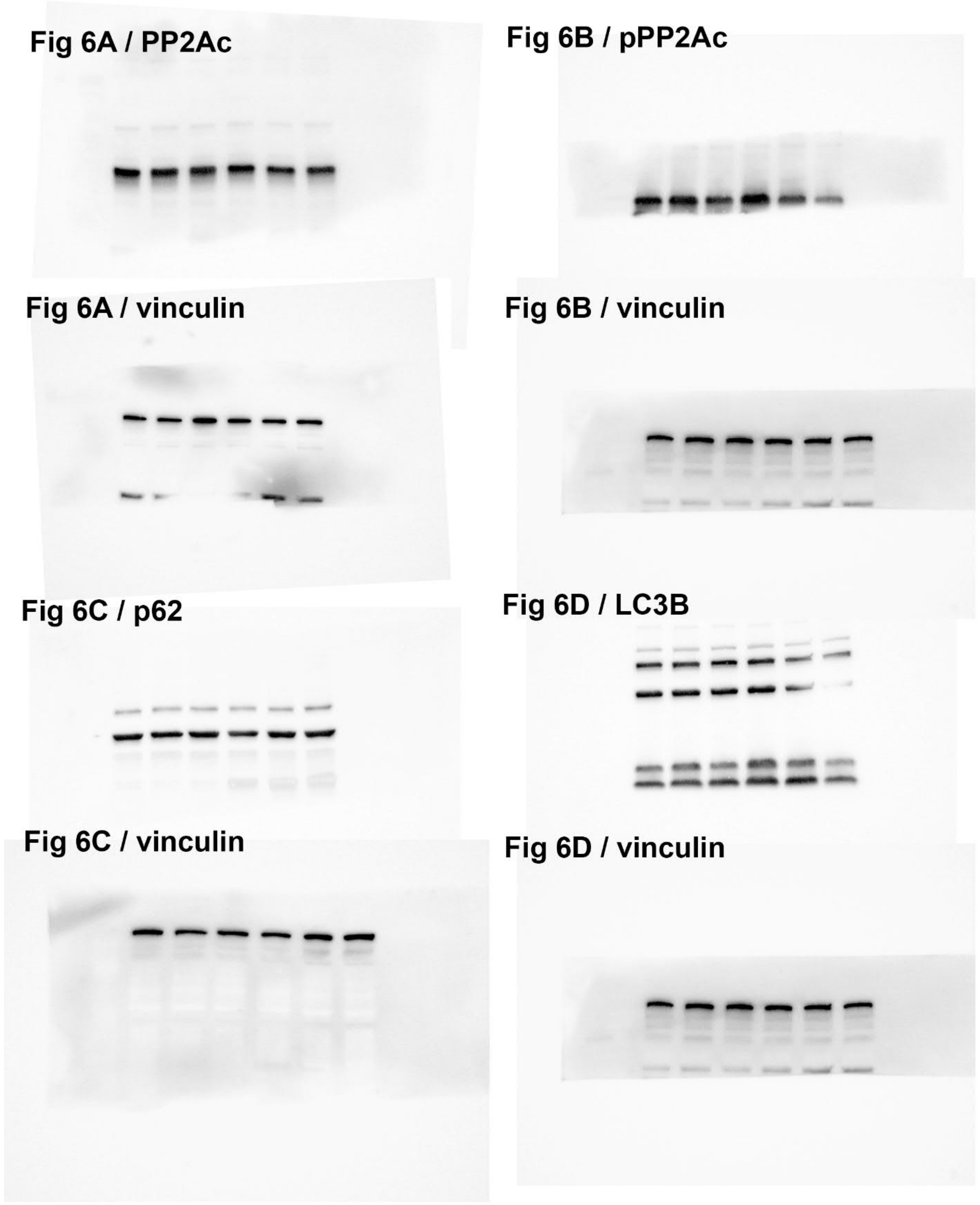

Figure S7. Uncut blots for Figure 6.

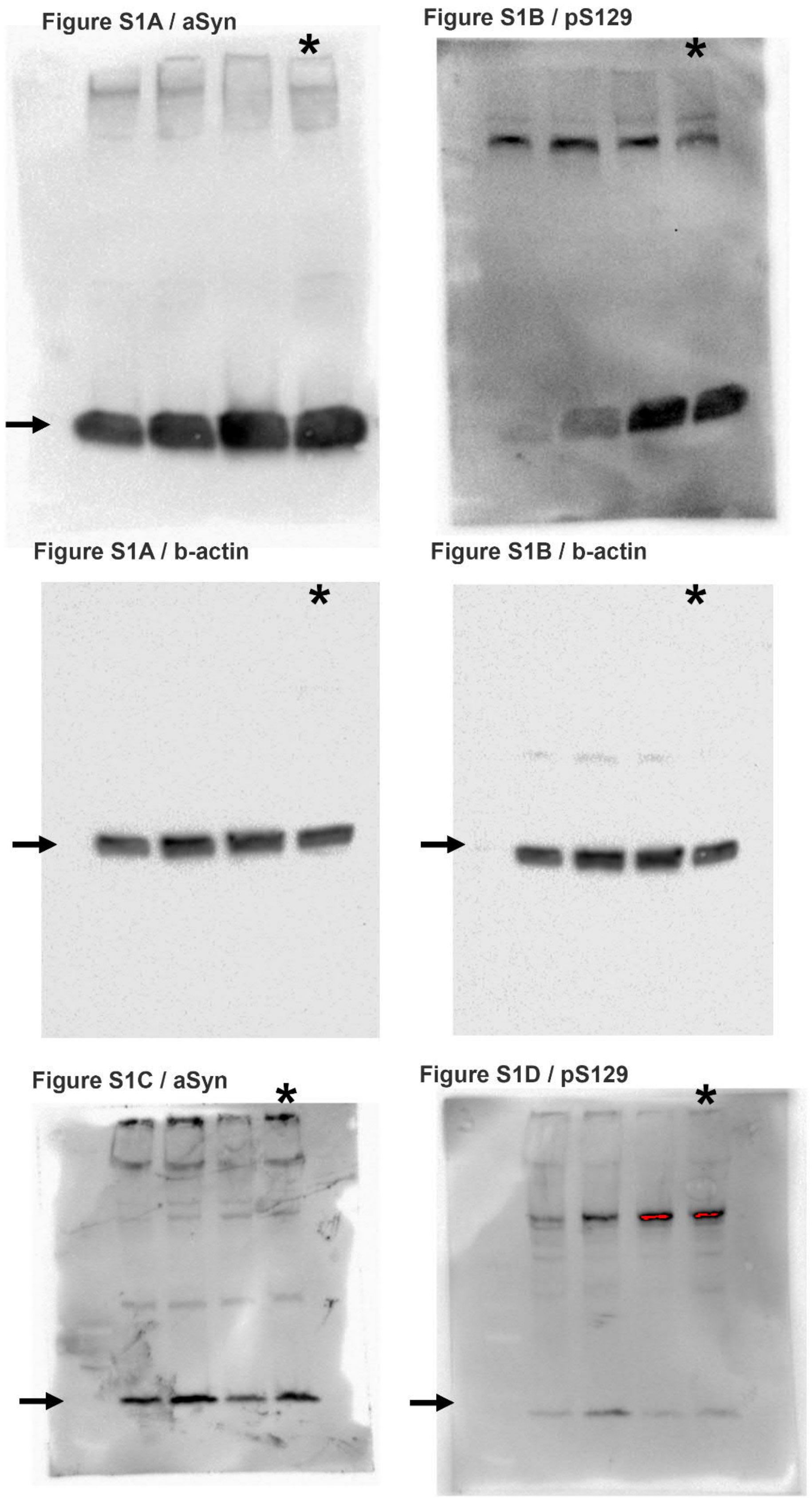

Figure S8. Uncut blots for Figure S1. Arrowhead marks the presented and analyzed band. * marks for experimental PREP inhibitor not published in this article (4th lane).

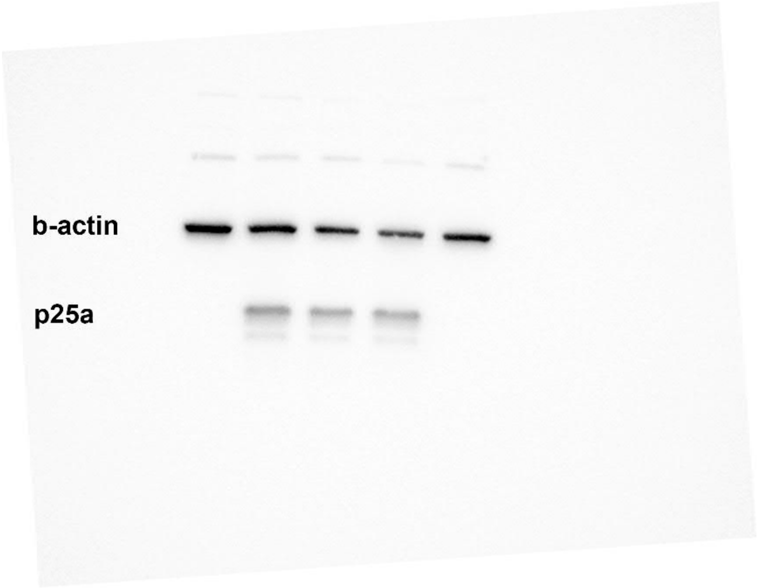

Figure S9. Uncut blots for Figure S3.
